# Driving cancer evolution

**DOI:** 10.7554/eLife.25431

**Published:** 2017-03-10

**Authors:** Devon M Fitzgerald, Susan M Rosenberg

**Affiliations:** 1 Department of Molecular and Human Genetics, Baylor College of Medicine, Houston, United States; 1 Department of Molecular and Human Genetics, Baylor College of Medicine, Houston, United Statessmr@bcm.edu; 2 Department of Biochemistry and Molecular Biology, Baylor College of Medicine, Houston, United States; 2 Department of Biochemistry and Molecular Biology, Baylor College of Medicine, Houston, United States; 3 Department of Molecular Virology and Microbiology, Baylor College of Medicine, Houston, United States; 3 Department of Molecular Virology and Microbiology, Baylor College of Medicine, Houston, United States; 4 Dan L Duncan Comprehensive Cancer Center, Baylor College of Medicine, Houston, United States; 4 Dan L Duncan Comprehensive Cancer Center, Baylor College of Medicine, Houston, United States

**Keywords:** genetic diversity, drug adaptability, intra-tumor heterogeneity, tumor evolution, tumor fitness, copy number alteration, Human

## Abstract

Tumor-growth-factor-beta signaling helps cancer cells to evolve and become resistant to drugs by down-regulating accurate DNA repair.

**Related research article** Pal D, Pertot A, Shirole NH, Yao Z, Anaparthy N, Garvin T, Cox H, Chang K, Rollins F, Kendall J, Edwards L, Singh VA, Stone GC, Schatz MC, Hicks J, Hannon GJ, Sordella R. 2017. TGF-β reduces DNA ds-break repair mechanisms to heighten genetic diversity and adaptability of CD44+/CD24− cancer cells. *eLife* **6**:e21615. doi: 10.7554/eLife.21615

Tumors can form when cells acquire mutations that allow them to grow and divide rapidly. Further mutations and the selection of successful cell clones allow the tumor to evolve and drive the progression of the disease with deadly consequences (reviewed by Gerlinger et al., 2014). Many tumors have unstable genomes with lots of small mutations and/or genome rearrangements, and this makes it more likely that the cancer will become progressively worse and evolve resistance to chemotherapy drugs (reviewed by Lee et al., 2016).

Historically, mutations were assumed to arise randomly, at constant rates. More recent discoveries indicate that cells and organisms can increase the rate at which they acquire new mutations (a process known as mutagenesis) when they activate cellular stress responses – that is, when they are stressed and poorly adapted to their environments (reviewed by Fitzgerald et al., 2017). Mutagenesis induced by stress has been predicted to speed up evolution (Ram and Hadany, 2012).

A signaling pathway known as tumor growth factor (TGF)-beta signaling promotes cell growth and is often up-regulated in cancer cells. Now, in eLife, Raffaella Sordella of Cold Spring Harbor Laboratory and colleagues – including Debjani Pal as first author – report that TGF-beta signaling in cancer cells temporarily down-regulates high-fidelity DNA repair, leading to populations of cells that are genetically diverse (Pal et al., 2017). These populations harbor more cells that are resistant to a broad range of chemotherapy drugs.

Pal et al. focused on a subpopulation of cells known as CD44+/CD24− cells, which arise randomly within many types of tumor and in cancer cell lines (reviewed by Polyak and Weinberg, 2009). They behave like stem cells thanks to the TGF-beta signaling pathway being continuously active, and are linked to drug-resistance, metastasis and other poor outcomes for patients (Shipitsin et al., 2007).

Cells generally use a process called homology-directed repair to mend double-stranded breaks in DNA. This process is mostly accurate and can heal DNA breaks without rearranging the chromosome. Pal et al. found that RNAs that encode proteins used in homology-directed repair and other types of DNA repair are less abundant in CD44+/CD24− cells. In these cells, DNA damage accumulates and genome re-arrangements occur, with the cells appearing to repair DNA breaks by other, less accurate, means.

TGF-beta signaling was necessary for homology-directed repair to be reduced in CD44+/CD24− cells, and sufficient for it to be reduced in other types of cells. Sequencing the genomes of individual cells demonstrated that TGF-beta signaling induces copy-number alterations – that is, insertions or deletions of gene-fragments to many neighboring genes – causing heritable genetic changes and populations of cells to become genetically diverse. Furthermore, Pal et al. used various cancer cell lines, primary tumor samples and published cancer genome datasets to demonstrate that TGF-beta signaling often decreases accurate DNA repair in cancer cells, which can cause copy-number alterations (Figure 1).

**Figure 1.**
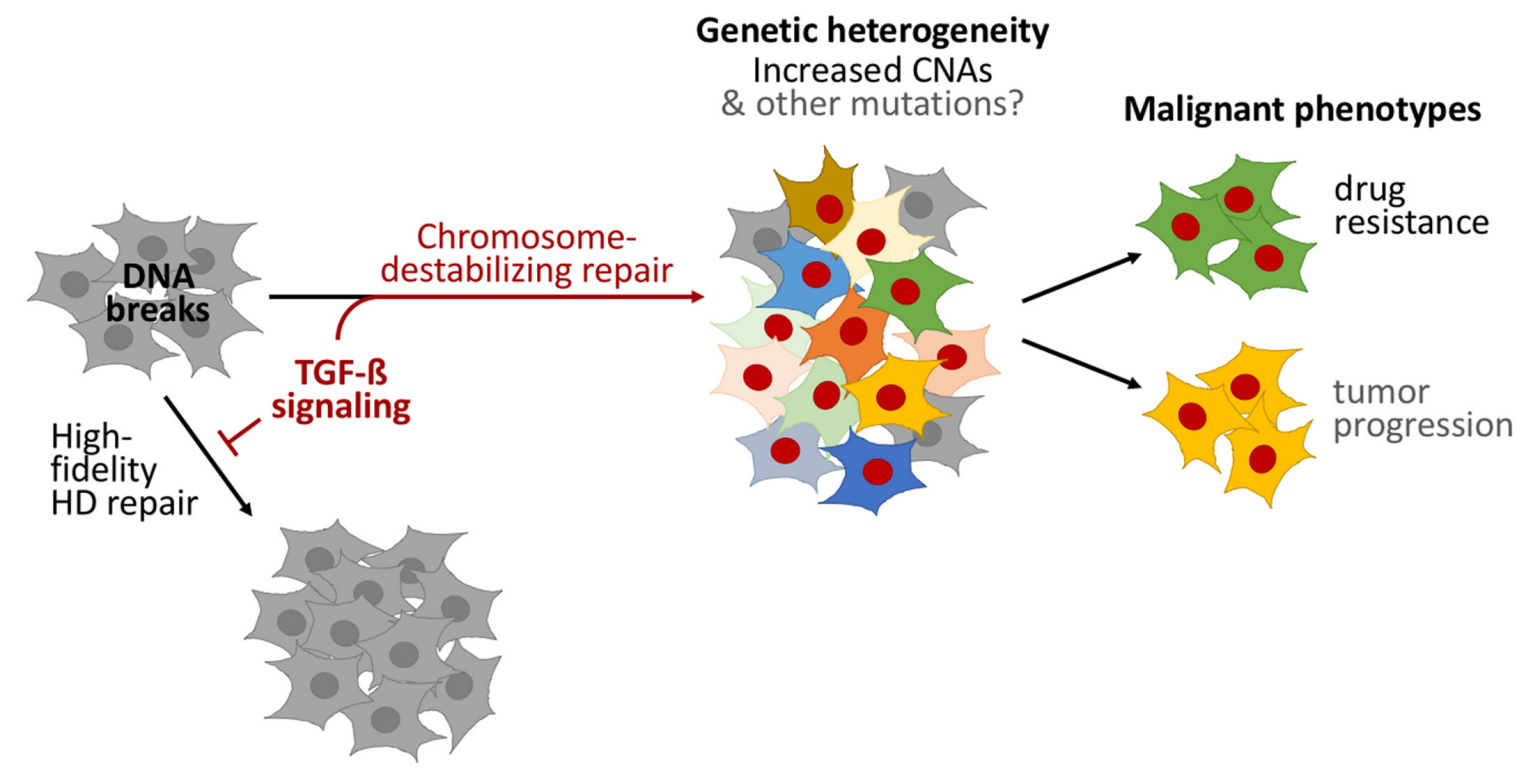
TGF-beta signaling promotes a form of DNA repair that accelerates cancer evolution. Cells generally repair spontaneous DNA breaks by a process called homology-directed (HD) repair (left). TGF-beta signaling down-regulates this process, which leads to the DNA breaks being repaired by other, less accurate, forms of repair that destabilize chromosomes and generate large DNA deletions and duplications known as copy-number alterations (CNAs). This leads to populations of cells that are genetically diverse (middle). Subpopulations of cancer cells known as CD44+/CD24− cells often have over-active TGF-beta signaling: this leads these cells to acquire high levels of CNAs and to rapidly evolve resistance to chemotherapy. Treating other cells with TGF-beta has a similar effect. Cells with red nuclei have undergone genetic changes; the other colors represent the resulting diverse cell characteristics.

A gene called *PMS1 –* which operates in another DNA repair process called mismatch-repair – was also less active in CD44+/CD24− cells. Mismatch repair corrects small errors in DNA replication, and more mutations accumulate when it is down-regulated (reviewed by Scanlon and Glazer, 2015; Fitzgerald et al., 2017). Although Pal et al. did not examine whether small mutations are increased, we suspect that, like copy-number alterations, TGF-beta may have increased the production of small mutations as well.

Does the genome instability caused by TGF-beta signaling drive the evolution of cancers? Pal et al. used a clever model of short-term exposure to TGF-beta to show that the heritable genomic changes that occurred, not the TGF-beta signaling itself, help the cancer cells to adapt. Cell populations previously exposed to TGF-beta harbored more cells that were resistant to three chemotherapy drugs than populations that had not been exposed. Although Pal et al. did not test the tendency of these cells to become more dangerous, the increased adaptability of cells exposed to TGF-beta probably speeds the progression of cancer as well (Figure 1).

The consequences are important. We previously suggested that a new class of “anti-evolvability” drugs should be developed to target the processes of cancer evolution, rather than targeting the products of evolution (that is, the disease characteristics; Rosenberg and Queitsch, 2014). Current anti-cancer drugs act to reduce the ability of cancer cells to grow and divide, and some induce mutagenesis (Fitzgerald et al., 2017). Inhibiting evolution itself might reduce the emergence of drug resistance.

Several stress-related proteins that promote mutagenesis and other mechanisms that change cell characteristics are promising targets for these new drugs (Rosenberg and Queitsch, 2014; Fitzgerald et al., 2017). The findings of Pal et al. suggest that proteins of the TGF-beta signaling pathway are potential targets for new “anti-evolvability” drugs that could be effective against many different types of cancer. Several drugs that target the TGF-beta pathway are already in clinical trials (reviewed by Herbertz et al., 2015).

This study also suggests the possibility that organisms may increase mutagenesis and evolution in the normal course of development. Programmed mutagenesis and evolution drive our adaptive immune systems, but might also underlie other complex developmental programs that require more flexibility and responsiveness than our genomes encode (discussed by Fitzgerald et al., 2017; Pal et al., 2017). TGF-beta signaling controls how wounds heal, certain aspects of embryo development, and how the nervous system forms. Future work may indicate that TGF-beta and other inducible mutagenesis responses allow multicellular complexity and flexibility in ways not yet appreciated.

## References

[bib1] Fitzgerald DM, Hastings PJ, Rosenberg SM (2017). Stress-induced mutagenesis: implications in cancer and drug resistance. Annual Review of Cancer Biology.

[bib2] Gerlinger M, McGranahan N, Dewhurst SM, Burrell RA, Tomlinson I, Swanton C (2014). Cancer: evolution within a lifetime. Annual Review of Genetics.

[bib3] Herbertz S, Sawyer JS, Stauber AJ, Gueorguieva I, Driscoll KE, Estrem ST, Cleverly AL, Desaiah D, Guba SC, Benhadji KA, Slapak CA, Lahn MM (2015). Clinical development of galunisertib (LY2157299 monohydrate), a small molecule inhibitor of transforming growth factor-beta signaling pathway. Drug Design, Development and Therapy.

[bib4] Lee JK, Choi YL, Kwon M, Park PJ (2016). Mechanisms and consequences of Cancer genome instability: lessons from genome sequencing studies. Annual Review of Pathology: Mechanisms of Disease.

[bib5] Pal D, Pertot A, Shirole NH, Yao Z, Anaparthy N, Garvin T, Cox H, Chang K, Rollins F, Kendall J, Edwards L, Singh VA, Stone GC, Schatz MC, Hicks J, Hannon GJ, Sordella R (2017). TGF-β reduces DNA ds-break repair mechanisms to heighten genetic diversity and adaptability of CD44+/CD24− cancer cells. eLife.

[bib6] Polyak K, Weinberg RA (2009). Transitions between epithelial and mesenchymal states: acquisition of malignant and stem cell traits. Nature Reviews Cancer.

[bib7] Ram Y, Hadany L (2012). The evolution of stress-induced hypermutation in asexual populations. Evolution.

[bib8] Rosenberg SM, Queitsch C (2014). Combating evolution to fight disease. Science.

[bib9] Scanlon SE, Glazer PM (2015). Multifaceted control of DNA repair pathways by the hypoxic tumor microenvironment. DNA Repair.

[bib10] Shipitsin M, Campbell LL, Argani P, Weremowicz S, Bloushtain-Qimron N, Yao J, Nikolskaya T, Serebryiskaya T, Beroukhim R, Hu M, Halushka MK, Sukumar S, Parker LM, Anderson KS, Harris LN, Garber JE, Richardson AL, Schnitt SJ, Nikolsky Y, Gelman RS, Polyak K (2007). Molecular definition of breast tumor heterogeneity. Cancer Cell.

